# Selection of rhythm intervention strategies in atrial fibrillation patients with cancer and efficacy and safety of catheter ablation

**DOI:** 10.3389/fcvm.2024.1506143

**Published:** 2024-11-22

**Authors:** Xin Wang, Xu Han, Xiaolei Yang, Tesfaldet Habtemariam Hidru, Chengfang Wang, Yunlong Xia, Ying Che

**Affiliations:** ^1^ Department of Ultrasound, First Affiliated Hospital of Dalian Medical University, Dalian, Liaoning, China; ^2^ Department of Ultrasound, The Affiliated Hospital of Innermongolia Medical University, Hohhot, China; ^3^ Health Management Center, First Affiliated Hospital of Dalian Medical University, Dalian, Liaoning, China; ^4^ Department of Cardiology, Institute of Cardiovascular Diseases, First Affiliated Hospital of Dalian Medical University, Dalian, Liaoning, China

**Keywords:** atrial fibrillation, cancer, catheter ablation, rhythm intervention, recurrence

## Abstract

**Background:**

The risk of comorbidity of cancer is increased in atrial fibrillation (AF) patients, which is a massive challenge for clinical management in cardiovascular settings. This study aimed to analyze whether cancer affects the decision of radiofrequency ablation and to explore the efficacy and safety of radiofrequency ablation in AF patients with cancer.

**Methods:**

We conducted a retrospective cohort study of patients who were first diagnosed AF and identified who were with cancer. The propensity score matching method was utilized to balance the differences between the cancer and non-cancer groups. Logistic regression analysis was used to study the related factors affecting the ablation of AF. Cox regression analysis was used to evaluate the effect of cancer on the recurrence of AF after radiofrequency ablation.

**Results:**

Among 9,159 patients who were first diagnosed AF, the prevalence of cancer was 4.48%. Cancer did not affect the decision of rhythm intervention in AF patients (*P* = 0.46). There was no significant difference in the incidence of perioperative complications, bleeding events, and embolization events between cancer and non-cancer groups (*P* = 1.000). The median follow-up time was 342 (293,866) days, and 45 patients had AF recurrence. Multivariable Cox regression showed no statistically significant relationship between concomitant cancer and AF recurrence after radiofrequency ablation (hazard ratio = 0.82, 95% confidence interval 0.36–1.83, *P* = 0.62).

**Conclusions:**

The combination of cancer did not affect the decision of patients to perform ablation therapy. Radiofrequency catheter ablation could be used as a strategy to maintain long-term sinus rhythm in patients with concomitant cancer without affecting AF recurrence.

## 1 Introduction

Atrial fibrillation (AF) is a prevalent arrhythmia, affecting around 1.5%–2% of the general population (1, 2). There exists a notable bidirectional relationship between cancer and AF, with cancer patients, especially older individuals or those with pre-existing cardiovascular risk factors, experiencing a significant increase in the incidence and prevalence of AF (3–5). Similarly, individuals with AF have an elevated risk of developing cancer compared to the general population (6, 7). Possible mechanisms include atrial remodeling due to proinflammatory states, autonomic nervous dysfunction, paraneoplastic syndrome, electrolyte abnormalities, and direct damage of cardiomyocytes due to anticancer therapy, surgery, or cancer metastasis (8–11). Patients with AF and cancer have a notably poorer prognosis compared to those with AF but without cancer, facing a 2-fold higher risk of thromboembolic events and a 6-fold higher risk of heart failure (12, 13).

Catheter ablation can be a viable first-line treatment for certain AF patients. This includes those who do not respond to antiarrhythmic drug therapy, have contraindications to medication, or have heart failure with a reduced ejection fraction (14). It's important to carefully consider rhythm intervention in AF patients with cancer, as the combination of antiarrhythmic drugs and specific targeted drugs may impact drug concentrations and increase the risk of arrhythmias. For instance, evidence revealed that tyrosine kinase inhibitors increase drug concentrations by impairing the cytochrome P-450 metabolic pathway or inhibiting P-glycoprotein-mediated transport (15). In addition, AF patients with cancer are more likely to develop arrhythmias such as QT prolongation and bradycardia, which further limits the use of antiarrhythmic drugs in this population (15). However, there may be underutilization of catheter ablation in AF patients with cancer due to concerns about the inflammatory status, use of potentially cardiotoxic drugs, and increased complication rates.

This study aims to evaluate the baseline characteristics and rhythm intervention choice for AF patients with cancer, determine the impact of cancer on the decision for catheter ablation, and assess the efficacy and safety of catheter ablation for AF in cancer patients.

## 2 Materials and methods

### 2.1 Study participants

We included patients who were hospitalized and first diagnosed with non-valvular AF in the Arrhythmia Center of the First Affiliated Hospital of Dalian Medical University from March 1, 2011, to July 1, 2023.

We initially recruited patients who were 18 years of age or older at the time of the hospitalization and had non-valvular AF as the primary diagnosis. We further excluded patients with other types of arrhythmias with indications for catheter ablation, such as atrial flutter, supraventricular tachycardia, preexcitation syndrome, premature ventricular complexes, and ventricular tachycardia and patients who underwent atrioventricular node ablation and pacemaker implantation during their current hospitalization. We also excluded patients with missing data for the essential covariates.

### 2.2 Ethics statement

This study was conducted in accordance with the Declaration of Helsinki of 2013. The requirement for informed consent was waived as this study was a retrospective analysis. Also, the study was approved by the institutional review board of the First Affiliated Hospital of Dalian Medical University (Reference No: PJ-KS-KY-2023–333).

### 2.3 Data collection and definition

We retrospectively retrieved clinical data from the electronic medical record system and YiDuloud Electronic Medical Surveillance Network database at First Affiliated Hospital of Dalian Medical University. Medical records of finally included patients were comprehensively reviewed by professional medical staff. The retrieved patient-related clinical data included demographic information (date of birth, gender, date of admission, date of discharge, etc.), physical examination indicators (systolic blood pressure, diastolic blood pressure), lifestyle (smoking history, drinking history), laboratory indexes (blood lipid, blood glucose, liver function, kidney function, electrolyte, blood routine, coagulation indicator, thyroid function, B-type natriuretic peptide), examination indicators (electrocardiogram, Holter, echocardiography), major cardiovascular diseases (hypertension, coronary heart disease, chronic heart failure, stroke, peripheral artery disease), major cardiovascular risk factors (type 2 diabetes, chronic kidney disease, hyperthyroidism, hypothyroidism, venous thromboembolism, and chronic obstructive pulmonary disease), and medication use (antihypertensive drugs, lipid-lowering drugs, antiplatelet drugs, hypoglycemic drugs, antiarrhythmic drugs and anticoagulant drugs).

For AF patients with cancer, we further collected cancer types (various solid tumors, hematological malignancies), anticancer treatments (surgery, chemotherapy, radiation therapy), and cancer status (active cancer and history of cancer). Active cancer included untreated cancer, metastatic cancer, and cancer undergoing treatment.

The choice of rhythm intervention treatment for AF patients is divided into two categories: drug-conservative treatment and radiofrequency catheter ablation treatment.

### 2.4 AF catheter ablation procedure

For all AF patients who underwent radiofrequency ablation, preoperative transesophageal echocardiography was performed to exclude left atrial thrombus and left atrial auricular thrombus. Pulmonary venous CT was performed to determine the pulmonary venous anatomy. The left atrial and pulmonary vein 3D models were reconstructed on Carto3 or Ensite NavX 3D electrical anatomical mapping system. Annular electrical isolation of bilateral pulmonary veins was performed until the pulmonary vein potential disappeared. If necessary, linear ablation, complex fractionated electrogram ablation, or ablation in low voltage areas was performed. Synchronous electrical cardioversion to sinus rhythm would be performed if the patients still had AF after these procedures. After 30 min of observation, the bidirectional ablation block was verified, and no tachycardia was detected by electrophysiological examination.

All AF patients who underwent radiofrequency ablation were followed up in the arrhythmia clinic in the 3rd month, 6th month, 12th month after discharge, and every year. The symptoms and medications were evaluated in the outpatient clinic, and the 24-h holter was completed. If patients had tachycardia-related symptoms such as palpitation, chest distress, and shortness of breath outside the outpatient follow-up time, a 12-lead electrocardiogram or 24-h Holter examination was performed to determine whether AF recurrence occurred. The blanking period was 3 months after ablation, and recurrence of AF was defined as any tachyarrhythmia of 30 s or more that occurred after the blanking period, including AF, atrial flutter, and atrial tachycardia.

### 2.5 Data analysis and statistical methods

Normally distributed numeric variables are presented as mean ± standard deviation, and an independent sample *t*-test was used to compare the two groups. Non-normally distributed continuous variables were represented by median (interquartile distance), and the Mann-Whitney *U*-test compared the two groups. Categorical variables were expressed by frequency (percentage), and group comparisons were made using the chi-square test.

Univariate and multivariable logistic regression analyses were used to study the related factors affecting the ablation of AF and to calculate their odds ratio (OR) and 95% confidence intervals (CI). Attention was paid to whether the combination of cancer had an impact on the choice of rhythm intervention.

The propensity score matching (PSM) method was utilized to balance the differences between the cancer and non-cancer groups. All covariates were indicators with significant differences between the two groups at baseline. The nearest number matching method was selected for 1:1 matching, and the Caliper value was set to 0.2 (i.e., the difference in propensity score value of each pair of successfully matched patients was ≤0.2). A total of 820 patients were included in the post-PSM analysis, including 410 patients with cancer and 410 patients without cancer, and univariate and multivariable logistic regression analysis was performed again after PSM. In a subgroup comprising 410 patients combined with cancer, we further compared the baseline characteristics between the radiofrequency ablation group and the conservative treatment group. Then, we conducted univariate and multivariable logistic regression analysis to explore related factors affecting the ablation of AF in the cancer population.

A total of 93 patients with cancer and 2,910 patients without cancer who underwent AF radiofrequency ablation were matched with propensity scores. The balance between the two groups was comparable except for coronary heart disease and hyperlipidemia. Finally, 92 patients with cancer and 92 patients without cancer were included in post-PSM analysis. Kaplan-Meier curve and log-rank test were used to compare the difference in AF recurrence rate between the two groups. The Cox proportional hazards regression model was used to evaluate the effect of cancer on the recurrence of AF after radiofrequency ablation, and the hazard ratio (HR) and 95% CI were calculated. Model 1 was adjusted for age and sex, and model 2 was adjusted for age, sex, types of AF, left atrial diameter (LAD), coronary artery disease, and hyperlipidemia.

Data analysis was carried out using SPSS version 25.0 and R version 4.3.0. All statistical analysis was two-sided; a *P*-value < 0.05 was considered statistically significant.

## 3 Results

### 3.1 Prevalence and distribution of cancer in AF patients

A total of 9,159 AF patients were included in this study, of which 410 were diagnosed with cancer, and the prevalence of cancer in AF patients was 4.48%. Among 410 AF patients with cancer, 42 had active cancer, accounting for 10.24%. Among them, 361 patients combined with one type of cancer, accounting for 88.05%, and 47 patients combined with two types of cancer, accounting for 11.46%. Two patients had three types of cancer, accounting for 0.49%. Breast, lung, thyroid, colon, gastric, and rectal cancers were the most common cancer types among AF patients hospitalized in the Arrhythmia Center of our hospital. This distribution of cancer types aligns with the findings previously published by our research team (16).

### 3.2 Comparison of baseline characteristics between AF patients with cancer and without cancer before PSM

As shown in Table 1, patients with cancer were older and had a lower proportion of males than those without cancer. In laboratory tests, cancer patients had lower levels of hemoglobin count, platelet count, total cholesterol, triglyceride, low-density lipoprotein cholesterol, high-density lipoprotein cholesterol and estimated glomerular filtration rate. Among the coagulation indicators, the fibrinogen level in AF patients with cancer was higher than that in the general AF population. In cardiovascular and related disease histories, patients with AF and cancer often suffered from hypertension, type 2 diabetes, chronic kidney disease, and venous thromboembolism. In contrast, the prevalences of coronary heart disease, heart failure, stroke, peripheral artery disease, dyslipidemia, hyperthyroidism, hypothyroidism, and chronic obstructive pulmonary disease were not significantly different between the two groups.

**Table 1:** Baseline characteristics between AF patients with cancer and without cancer before PSM.

Variables	Total (*n* = 9,159)	No Cancer (*n* = 8,749)	Cancer (*n* = 410)	*P-*value	SMD
Age, years	65.72 (11.35)	65.46 (11.38)	71.18 (9.18)	<0.001	0.623
Male, *n* (%)	5,328 (58.17)	5,117 (58.49)	211 (51.46)	0.005	−0.141
Hospital stays, days	6.28 (3.96)	6.28 (3.95)	6.19 (4.06)	0.64	−0.023
Smoking, *n* (%)	1,790 (19.77)	1,725 (19.96)	65 (15.89)	0.044	−0.106
Drinking, *n* (%)	1,240 (13.7)	1,191 (13.78)	49 (11.98)	0.30	−0.051
SBP, mmHg	134.17 (19.06)	134.14 (19.07)	134.70 (18.85)	0.57	0.029
DBP, mmHg	81.47 (16.58)	81.52 (16.72)	80.43 (13.23)	0.19	−0.082
Hemoglobin count, g/L	140.79 (16.43)	141.08 (16.29)	134.43 (17.98)	<0.001	−0.370
Platelet count, ×109/L	200.38 (50.54)	200.68 (50.24)	193.89 (56.20)	0.008	−0.121
NLR	1.86 (1.46, 2.40)	1.86 (1.46, 2.39)	1.86 (1.59, 2.67)	0.002	0.099
TC, mmol/L	4.57 (0.77)	4.58 (0.77)	4.47 (0.75)	0.005	−0.146
TG, mmol/L	1.43 (0.71)	1.44 (0.72)	1.35 (0.53)	0.015	−0.164
LDL-C, mmol/L	2.58 (0.56)	2.58 (0.56)	2.49 (0.55)	0.002	−0.163
HDL-C, mmol/L	1.21 (0.24)	1.21 (0.24)	1.18 (0.24)	0.021	−0.113
ALT, IU/L	28.63 (48.69)	28.85 (49.71)	24.03 (14.42)	0.050	−0.334
AST, IU/L	26.40 (47.03)	26.50 (48.06)	24.35 (10.98)	0.37	−0.196
eGFR, ml/min/1.73m^2^	87.20 (23.56)	87.34 (23.47)	84.08 (25.34)	0.011	−0.129
SUA, μmol/L	364.03 (93.86)	364.40 (93.96)	356.11 (91.38)	0.080	−0.091
K^+^, mmol/L	4.02 (0.39)	4.02 (0.39)	4.05 (0.44)	0.080	0.083
Na^+^, mmol/L	141.91 (2.66)	141.92 (2.64)	141.54 (3.08)	0.013	−0.125
Mg^2+^, mmol/L	0.93 (0.09)	0.93 (0.09)	0.92 (0.09)	0.20	−0.082
FBG, mmol/L	5.77 (1.81)	5.76 (1.81)	5.90 (1.79)	0.26	0.075
Coagulation indicators					
TT, s	21.37 (19.17)	21.38 (19.20)	21.23 (18.63)	0.89	−0.008
PT, s	13.61 (6.30)	13.61 (6.34)	13.48 (5.33)	0.69	−0.026
APTT, s	28.20 (9.40)	28.12 (7.27)	30.00 (28.44)	0.21	0.066
Fib, g/L	2.84 (0.69)	2.83 (0.68)	2.98 (0.87)	<0.001	0.173
TSH, mIU/L	2.69 (3.33)	2.67 (2.98)	3.12 (7.64)	0.28	0.060
BNP, ng/L	189.83 (264.05)	188.94 (266.06)	208.77 (216.19)	0.14	0.092
Echocardiographic parameters					
LAD, mm	40.58 (5.97)	40.58 (5.99)	40.55 (5.64)	0.94	−0.005
LVEDD, mm	48.14 (5.71)	48.16 (5.73)	47.64 (5.29)	0.14	−0.098
LVEF,%	55.18 (7.56)	55.16 (7.61)	55.61 (6.46)	0.33	0.069
Previous history, *n* (%)					
HTN	5,181 (56.57)	4,918 (56.21)	263 (64.15)	0.002	0.165
CHD	3,321 (36.26)	3,186 (36.42)	135 (32.93)	0.15	−0.074
HF	2,296 (25.07)	2,179 (24.91)	117 (28.54)	0.097	0.080
Stroke	945 (10.32)	897 (10.25)	48 (11.71)	0.34	0.045
PAD	157 (1.71)	154 (1.76)	3 (0.73)	0.12	−0.121
Dyslipidemia	5,624 (61.4)	5,376 (61.45)	248 (60.49)	0.70	−0.020
T2DM	1,846 (20.16)	1,739 (19.88)	107 (26.10)	0.002	0.142
CKD	851 (9.29)	793 (9.06)	58 (14.15)	<0.001	0.146
Hyperthyroidism	182 (1.99)	173 (1.98)	9 (2.20)	0.76	0.015
Hypothyroidism	119 (1.3)	112 (1.28)	7 (1.71)	0.46	0.033
VTE	28 (0.31)	24 (0.27)	4 (0.98)	0.04	0.071
COPD	68 (0.74)	65 (0.74)	3 (0.73)	1.000	−0.001
Types of AF, *n* (%)				0.78	
PaAF	5,277 (57.62)	5,038 (57.58)	239 (58.29)		0.014
PeAF	3,882 (42.38)	3,711 (42.42)	171 (41.71)		−0.014
Antiarrhythmic drugs, *n* (%)				0.038	
Amiodarone	2,728 (29.78)	2,629 (30.05)	99 (24.15)		−0.138
Propafenone	1,419 (15.49)	1,351 (15.44)	68 (16.59)		0.031
*β*-blocker	5,012 (54.72)	4,769 (54.51)	243 (59.27)		0.097
Anticoagulants, *n* (%)				0.35	
Warfarin	4,741 (51.76)	4,538 (51.87)	203 (49.51)		−0.047
NOACs	4,418 (48.24)	4,211 (48.13)	207 (50.49)		0.047
AF radiofrequency ablation, *n* (%)	3,003 (32.79)	2,910 (33.26)	93 (22.68)	<0.001	−0.253

Abbreviations: AF, atrial fibrillation; ALT, alanine transaminase; APTT, activated partial thromboplastin time; AST, aspartate aminotransferase; BNP, B-type natriuretic peptide; CHD, coronary heart disease; CKD, chronic kidney disease; COPD, chronic obstructive pulmonary disease; DBP, diastolic blood pressure; eGFR, estimated glomerular filtration rate; FBG, fasting blood glucose; Fib, plasma fibrinogen; HDL-C, high-density lipoprotein cholesterol; HF, heart failure; HTN, hypertension; LAD, left atrial diameter; LDL-C, low-density lipoprotein cholesterol; LVEDD, left ventricular end diastolic diameter; LVEF, left ventricular ejection fraction; NLR, neutrophil to lymphocyte ratio; NOACs, non-vitamin K antagonist oral anticoagulants; PaAF, paroxysmal atrial fibrillation; PAD, peripheral arterial disease; PeAF, persistent atrial fibrillation; PSM, Propensity score matching; PT, prothrombin time; SBP, systolic blood pressure; SMD, standardized mean difference; SUA, serum uric acid; T2DM, type2 diabetes mellitus; TC, total cholesterol; TG, triglyceride; TT, thrombin time; TSH, thyroid stimulating hormone; VTE, venous thromboembolism.

The two groups had no significant difference in the clinical type of AF (paroxysmal AF vs. persistent AF). More than half of the total population had paroxysmal AF, and the prevalence was 58.29% in cancer patients and 57.58% in the non-cancer population. In antiarrhythmic drug use, AF patients with cancer were less likely to use amiodarone and more likely to choose *β*-blockers. In the overall population, slightly more patients chose warfarin than Non-vitamin K antagonist oral anticoagulants, but there was no significant difference. 50.49% of cancer patients chose Non-vitamin K antagonist oral anticoagulants, and 51.87% of non-cancer patients chose warfarin, but there was no significant difference in the choice of anticoagulant drugs between the two groups. In the context of patients' rhythm intervention decision-making, the percentage of patients with AF and cancer who underwent radiofrequency ablation was notably lower at 22.68% compared to those without cancer at 33.26%, showing a statistically significant difference (*P* < 0.001).

### 3.3 Factors associated with ablation in AF patients before PSM

Table 2 shows the factors affecting the choice of rhythm intervention in the AF population before PSM. The candidate variables included in the logistic regression were related to patients' decision to perform radiofrequency ablation in clinical practice. The multivariable regression analysis showed that patients with older age (OR = 0.95, 95%CI 0.94–0.96, *P* < 0.001), higher thyroid stimulating hormone (OR = 0.97, 95%CI 0.95–0.99, *P* = 0.044), larger LAD (OR = 0.95, 95%CI 0.94–0.97, *P* < 0.001), heart failure (OR = 0.53, 95%CI 0.44–0.64, *P* < 0.001), chronic kidney disease (OR = 0.57, 95%CI 0.43–0.74, *P* < 0.001) and persistent AF (OR = 0.81, 95CI 0.70–0.93, *P* = 0.003) were less likely to undergo ablation. The presence of cancer did not have a statistically significant effect on the decision to perform radiofrequency ablation in patients with AF, according to the results of multivariable logistic regression analysis (OR = 0.77, 95%CI 0.56–1.05, *P* = 0.100).

**Table 2:** Factors associated with ablation in AF patients before PSM.

Variables	Univariable analysis		Multivariable analysis	
	OR (95%CI)	*P*-value	OR (95%CI)	*P*-value
Age	0.94 (0.94–0.95)	<0.001	0.95 (0.94–0.96)	<0.001
TSH	0.96 (0.94–0.98)	<0.001	0.97 (0.95–0.99)	0.044
LAD	0.92 (0.91–0.93)	<0.001	0.95 (0.94–0.97)	<0.001
Male	1.27 (1.16–1.39)	<0.001	1.08 (0.94–1.23)	0.27
HTN	0.68 (0.62–0.74)	<0.001	1.08 (0.95–1.24)	0.25
CHD	0.64 (0.58–0.70)	<0.001	0.91 (0.79–1.04)	0.17
HF	0.26 (0.23–0.30)	<0.001	0.53 (0.44–0.64)	<0.001
Stroke	0.58 (0.50–0.68)	<0.001	0.89 (0.72–1.10)	0.28
PAD	0.56 (0.38–0.82)	0.003	0.56 (0.30–1.03)	0.06
T2DM	0.73 (0.65–0.82)	<0.001	0.96 (0.82–1.13)	0.63
CKD	0.27 (0.22–0.33)	<0.001	0.57 (0.43–0.74)	<0.001
VTE	0.34 (0.12–0.98)	0.046	0.82 (0.24–2.74)	0.74
PeAF	0.54 (0.49–0.59)	<0.001	0.81 (0.70–0.93)	0.003
Cancer	0.59 (0.47–0.74)	<0.001	0.77 (0.56–1.05)	0.10

Abbreviations: see Table 1; OR, odds ratio.

### 3.4 Factors associated with ablation in AF patients after PSM

After 1:1 matching, 410 patients with cancer and 410 patients without cancer were included in the post-matching analysis. Supplementary Material Table S1 shows the baseline characteristics of the two groups. After PSM, the two groups were well balanced, except for coronary heart disease. After matching, there was no significant difference in the proportion of AF ablation in AF patients with and without cancer (22.68% vs. 24.88%, *P* = 0.460).

Supplementary Material, Table S2 shows the univariate and multivariable logistic regression analysis of 820 AF patients after matching to evaluate the factors affecting the AF ablation decision. The variables with a *P*-value of less than 0.05 in the univariate regression were exclusively included in the multivariable regression analysis. The analysis indicated that patients with older age (OR = 0.94, 95%CI 0.90–0.97, *P* < 0.001), larger LAD (OR = 0.94, 95%CI 0.90–0.99, *P* = 0.018) and diabetes (OR = 0.56, 95%CI 0.31–0.99, *P* = 0.046) exhibited a reduced likelihood of undergoing AF radiofrequency ablation.

### 3.5 Comparison of baseline characteristics between cancer patients who receive ablation and those who do not receive ablation

We conducted a subgroup analysis to explore further the differences in the selection of rhythm intervention in AF patients with cancer. The patients with cancer were divided into two groups according to the choice between conservative treatment and radiofrequency ablation at the time of hospitalization. The baseline characteristics between the two groups are shown in Supplementary material Table S3. Most cancer patients who underwent ablation were of the paroxysmal AF type (68.82% vs. 55.21%, *P* = 0.019). Amiodarone was often used as an antiarrhythmic drug for them (64.52% vs. 12.30%, *P* < 0.001), while cancer patients with conservative treatment preferred *β*-blockers (71.92% vs. 16.13%, *P* < 0.001). There was no significant difference in cancer activity between the two groups (9.68% vs. 10.41%, *P* = 0.838), and there was no significant difference in anticancer treatment (including surgery, chemotherapy, and radiotherapy) between the two groups.

### 3.6 Factors associated with ablation in AF patients with cancer

Table 3 shows the univariate and multivariable logistic regression analysis results in 410 patients with AF and cancer. Based on the multivariable regression analysis findings, it was revealed that only younger patients (OR = 0.93, 95%CI 0.90–0.96, *P* < 0.001) were more likely to undergo AF radiofrequency ablation in AF patients with cancer.

**Table 3:** Factors associated with ablation in AF patients with cancer.

Variables	Univariable analysis		Multivariable analysis	
	OR (95%CI)	*P-*value	OR (95%CI)	*P*-value
Age	0.92 (0.89–0.95)	<0.001	0.93 (0.90–0.96)	<0.001
Hb	1.01 (0.99–1.02)	0.23		
PLT	1.00 (1.00–1.01)	0.20		
TSH	0.91 (0.81–1.04)	0.16		
LAD	0.97 (0.92–1.02)	0.21		
Male	0.61 (0.38–0.97)	0.037	0.68 (0.41–1.14)	0.15
HTN	0.72 (0.45–1.15)	0.17		
CHD	0.48 (0.28–0.83)	0.009	0.62 (0.35–1.12)	0.11
HF	0.37 (0.20–0.68)	0.001	0.62 (0.31–1.24)	0.18
DM	0.56 (0.32–1.01)	0.053		
Stroke	0.55 (0.24–1.27)	0.16		
CKD	0.28 (0.11–0.73)	0.009	0.37 (0.14–1.00)	0.051
PAD	1.71 (0.15–19.09)	0.66		
Active cancer	0.92 (0.42–2.00)	0.84		
surgery	2.41 (0.83–7.02)	0.11		
chemotherapy	1.00 (0.48–2.12)	0.99		
radiotherapy	0.59 (0.17–2.05)	0.41		
PeAF	0.56 (0.34–0.91)	0.02	0.75 (0.43–1.30)	0.31

Abbreviations: see Table 1; CI, confidence interval; Hb, hemoglobin; OR, odds ratio; PLT, platelet.

### 3.7 Efficacy and safety of catheter ablation for AF in patients with and without cancer

Following a 1:1 matching process, 92 AF patients with cancer and 92 patients without cancer who received radiofrequency ablation were included in the post-matching analysis and subsequent follow-up. Supplementary Table S4 shows the baseline characteristics of the two groups. After PSM, the balance of the two groups was comparable except for coronary heart disease and dyslipidemia.

A total of 184 AF patients were followed up for a median of 342 (293,866) days. The recurrence rate was 24.5% (45/184), including 23.9% (22/184) in patients with cancer and 25.0% (23/184) in patients without cancer. During the follow-up period, there were 1 case of cerebral thromboembolism (1.09%) and 4 cases of mucocutaneous hemorrhage (1.09%) in AF patients with cancer. In AF patients without cancer, there were 1 case of cerebral thromboembolism (1.09%) and 4 cases of mucocutaneous hemorrhage (4.35%) as well. In addition, pericardial tamponade occurred in 1 patient with cancer during the perioperative period, and there were no severe complications in typical AF patients. However, the two groups had no statistically significant differences (*P* = 1.000).

**Figure 1:**
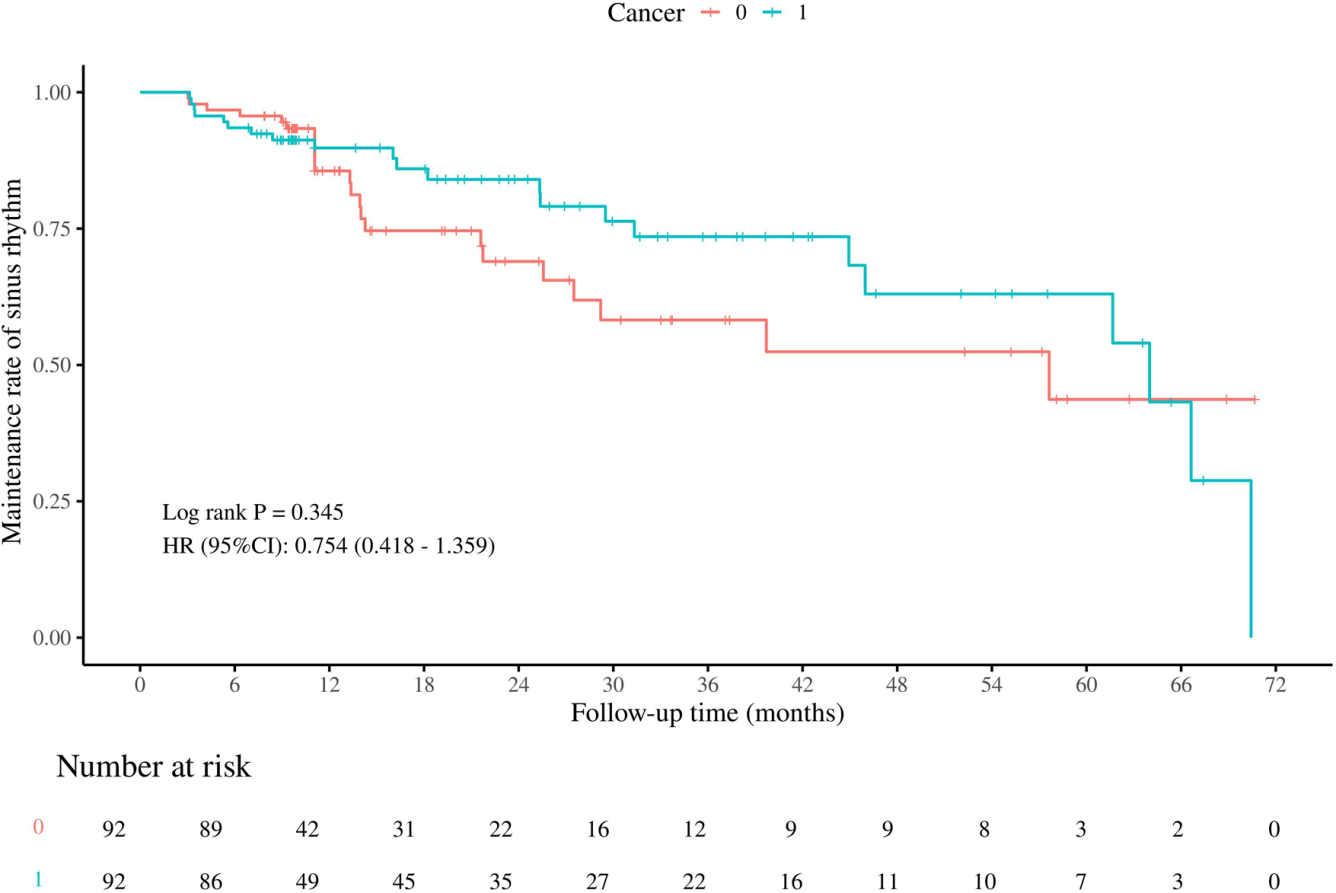
Maintenance rate of sinus rhythm between AF patients with and without cancer after ablation. Figure shows no significant difference in the maintenance rate of sinus rhythm after radiofrequency ablation between the patients with and without cancer (*P* = 0.345).

Figure 1 shows the Kaplan-Meier curve for comparing the maintenance rate of sinus rhythm between the two groups. The log-rank test showed no significant difference in the maintenance rate of sinus rhythm after radiofrequency ablation between the patients with and without cancer (*P* = 0.345).

In model 1, after adjusting for age and sex, cancer was not a risk factor for AF recurrence after radiofrequency ablation (HR = 0.73, 95%CI 0.40–1.32, *P* = 0.30). In model 2, after adjusting for age, sex, AF type, LAD, coronary heart disease, and dyslipidemia, cancer was still not significantly associated with AF recurrence after radiofrequency ablation (HR = 0.82, 95%CI 0.36–1.83, *P* = 0.62) (Table 4).

**Table 4:** The effect of cancer on the recurrence of AF after radiofrequency ablation.

	Recurrence cases	Model 1		Model 2	
		HR (95% CI)	*P-*value	HR (95% CI)	*P-*value
No cancer	23 (25.0%)	Reference		Reference	
Cancer	22 (23.9%)	0.73 (0.40–1.32)	0.30	0.82 (0.36–1.83)	0.62

Model 1: adjust for age and sex.

Model 2: adjust for age, sex, AF type, left atrial diameter, coronary heart disease and dyslipidemia.

Abbreviations: AF, atrial fibrillation; CI, confidence interval; HR, hazard ratio.

## 4 Discussion

This study revealed that the presence of cancer did not influence the decision-making process for rhythm intervention in AF patients, both before and after PSM. Notably, in AF patients with cancer, the specific cancer activity and anticancer treatment did not impact the choice of rhythm intervention. Furthermore, the co-existence of cancer did not affect the recurrence of AF following radiofrequency ablation.

### 4.1 The safety and effectiveness of radiofrequency ablation in AF patients with cancer

The safety and effectiveness of radiofrequency ablation in AF patients with cancer is an important consideration (17–19). Individuals with both AF and cancer face a heightened risk of severe complications, such as systemic embolism and heart failure (12). Therefore, effectively managing AF in high-risk cancer patients is crucial. While catheter ablation is the primary treatment for restoring normal heart rhythm in AF, there is a lack of comprehensive studies involving cancer patients in major clinical trials like CABANA and CASTLE AF. This gap has left uncertainties about the safety and efficacy of catheter ablation for AF in the cancer population. However, the 2022 ESC guidelines on cardio-oncology suggest that the potential for AF ablation should be discussed in specific cases, taking into account factors such as heart failure, symptoms, cancer status, and prognosis in a multidisciplinary team setting (20, 21). Concerns about complications during the perioperative period and AF recurrence after ablation are also common when considering catheter ablation for AF in cancer patients.

In a retrospective cohort study, researchers found that cancer patients undergoing catheter ablation for AF had a significantly higher risk of in-hospital death, major bleeding, and pulmonary embolism after PSM when compared to those without cancer (22). Giustozzi et al. also discovered that the occurrence of clinically significant bleeding after AF catheter ablation was higher in cancer survivors than in the general population during a 1-month follow-up (23). Agarwal et al. reported that 11.7% of AF patients undergoing catheter ablation had a history of cancer, and 2.8% had active cancer. When compared with patients without cancer, those with active cancer were found to have an increased risk of perioperative complications, as well as 30-day, 90-day, and 180-day all-cause readmissions and bleeding-related readmissions. Additionally, there were no significant differences in perioperative complications and all-cause readmissions between patients with a history of cancer and those without cancer (24). Interestingly, a study conducted by Wu et al. demonstrated that active cancer, but not a history of cancer, was associated with an increased risk of in-hospital mortality when adjusting for confounders (25). These findings shed light on the unique challenges faced by AF patients with a history of or active cancer.

What is more, Ganatra et al. found that 12 months after ablation, having a history of cancer or undergoing cancer-related treatment did not independently increase the risk of AF recurrence. The study also showed that there was no disparity in safety outcomes between AF patients with a history of cancer and those without. The researchers concluded that catheter ablation was a safe and effective treatment for AF patients with a cancer history and those exposed to anthracyclines and/or thoracic radiation (26). Eitel et al. utilized PSM to compare 70 AF patients with cancer to 70 AF patients without cancer who underwent cryo-balloon pulmonary vein isolation. Their findings revealed no significant variance in surgical complications and the maintenance of sinus rhythm at 12 months post-ablation between the two groups, indicating the safety and effectiveness of cryo-balloon pulmonary vein isolation for treating symptomatic AF in both cancer and non-cancer patients (27).

Since different cancers have different risk profiles, the safety and the efficacy of radiofrequency ablation might be dependent on the type of cancer a patient has. Peng et al. observed that hematological malignancy was associated with a high risk of AF recurrence compared with the matched non-cancer group. Whereas types of cancer, including breast, lung, prostate, thyroid, urinary, gastrointestinal and genital cancer, did not significantly affect AF recurrence (28). Thotamgari et al. assessed the outcomes and peri-procedural safety of catheter ablation for AF in patients with certain types of cancer. They reported that breast cancer had the highest all-cause in-hospital mortality whereas hematological cancers had the highest risk of major bleeding. Respiratory cancers had lowest rates of home discharges and higher risk of pulmonary embolism whereas higher rates of acute kidney injury were observed with urinary tract malignancies (22). Due to the sample size, we cannot determine the efficacy and safety of AF radiofrequency ablation in patients with certain cancer types. Further studies are needed to evaluate the differences in outcomes based on type of cancers.

The present study demonstrated that, even after adjusting for confounding factors, cancer did not influence the collaborative decision-making process between doctors and patients when choosing a rhythm intervention for AF. Furthermore, the present study found that cancer did not significantly impact the recurrence of AF following radiofrequency ablation. The efficacy and safety of ablation in AF patients with cancer were found to be comparable to those in the general AF population with similar baseline characteristics. These findings were consistent with previous studies conducted at our center (29). The study also showed that cancer was not a significant risk factor for AF recurrence after catheter ablation. Our hospital has established a specialized cardio-oncology diagnosis and treatment center and has been at the forefront of promoting cardio-oncology nationally. Due to our extensive experience in treating cancer patients with different cardiovascular and cerebrovascular conditions, our thorough expertise in managing this specific group of patients may influence the decision to conduct catheter ablation. Therefore, it's clear that the absence of a statistically significant association between cancer and the choice of rhythm intervention in AF patients was noted in this study.

### 4.2 Comprehensive management of AF patients with cancer

When managing the ventricular rate and rhythm control of AF in cancer patients, it's important to consider several factors. Rhythm-control medications may lead to QT interval prolongation and can interact with chemotherapeutic agents. Especially when the cancer treatment itself triggers AF episodes, these drugs may have little effect in such cases. For cancer patients, it is recommended to use *β*-blockers for ventricular rate control, mainly when there is potential cancer-related heart function impairment. Drugs like diltiazem and verapamil should be avoided due to their interactions with other medications and negative inotropic effects (30). There are complexities involved in managing new-onset AF in cancer patients, especially in assessing the risk of stroke or systemic embolism. According to the 2022 ESC guidelines on cardio-oncology, the CHA2DS2-VASc score should be utilized for risk stratification (31), although it hasn't been widely validated in the cancer population (32). It's important to note that this score was not designed to identify high-risk patients, but to identify low-risk patients who may not require anticoagulation. Consequently, the cardio-oncology guidelines offer a structured approach to anticoagulation for AF in patients with cancer, taking into account thrombotic risk, bleeding risk, drug interactions, and patient access and preferences. In some cases, left atrial appendage occlusion may be considered for stroke prevention in cancer patients with AF and contraindications for long-term anticoagulation, provided that they have a life expectancy of more than 12 months (14, 33).

### 4.3 Limitations

This study was a retrospective cohort study, which may be subject to bias. The treatment choice for patients with AF and cancer in our study may have been influenced by the development of cardio-oncology in our center. In other medical centers, the decision to perform catheter ablation might be impacted by the lack of experience in treating this particular group. It's important to acknowledge that this study was conducted at a single center with a limited sample size. As a result, further multi-center studies with larger sample size are warranted to ascertain whether there are variations in outcomes for patients with different types of cancer who undergo radiofrequency ablation for AF and to assess the impact on postoperative recurrence.

## 5 Conclusions

In our center, the presence of cancer did not influence the decision to perform ablation among AF patients. Additionally, in AF patients with cancer, factors such as the status of the cancer (active or stable) and the type of anti-cancer treatment (surgery, chemotherapy, radiotherapy) did not impact the decision to perform ablation. Furthermore, the presence of cancer did not affect the recurrence of AF after radiofrequency catheter ablation. As a result, radiofrequency catheter ablation could be considered as one of the strategies for achieving long-term maintenance of normal heart rhythm in AF patients with cancer.

## Data Availability

The raw data supporting the conclusions of this article will be made available by the authors, without undue reservation.

## References

[1] Paterson DI Wiebe N Cheung WY Mackey JR Pituskin E Reiman A Incident cardiovascular disease among adults with cancer: a population-based cohort study. J Am Coll Cardiol CardioOnc. (2022) 4(1):85–94. 10.1016/j.jaccao.2022.01.100 PMC9040097 35492824

[2] Shi S Tang Y Zhao Q Yan H Yu B Zheng Q Prevalence and risk of atrial fibrillation in China: a national cross-sectional epidemiological study. Lancet Reg Health West Pac. (2022) 23:100439. 10.1016/j.lanwpc.2022.100439 35800039 PMC9252928

[3] Chu G Versteeg HH Verschoor AJ Trines SA Hemels MEW Ay C Atrial fibrillation and cancer—an unexplored field in cardiovascular oncology. Blood Rev. (2019) 35:59–67. 10.1016/j.blre.2019.03.005 30928168

[4] Menichelli D Vicario T Ameri P Toma M Violi F Pignatelli P Cancer and atrial fibrillation: epidemiology, mechanisms, and anticoagulation treatment. Prog Cardiovasc Dis. (2021) 66:28–36. 10.1016/j.pcad.2021.04.004 33915139

[5] Erichsen R Christiansen CF Mehnert F Weiss NS Baron JA Sorensen HT. Colorectal cancer and risk of atrial fibrillation and flutter: a population-based case-control study. Intern Emerg Med. (2012) 7(5):431–8. 10.1007/s11739-011-0701-9 21968511

[6] Guha A Dey AK Jneid H Ibarz JP Addison D Fradley M. Atrial fibrillation in the era of emerging cancer therapies. Eur Heart J. (2019) 40(36):3007–10. 10.1093/eurheartj/ehz649 31541552 PMC6933869

[7] Vinter N Christesen AMS Fenger-Gron M Tjonneland A Frost L. Atrial fibrillation and risk of cancer: a danish population-based cohort study. J Am Heart Assoc. (2018) 7(17):e009543. 10.1161/JAHA.118.009543 30371150 PMC6201425

[8] Leiva O AbdelHameid D Connors JM Cannon CP Bhatt DL. Common pathophysiology in cancer, atrial fibrillation, atherosclerosis, and thrombosis: JACC: cardioOncology state-of-the-art review. J Am Coll Cardiol CardioOnc. (2021) 3(5):619–34. 10.1016/j.jaccao.2021.08.011 34988471 PMC8702799

[9] Farmakis D Parissis J Filippatos G. Insights into onco-cardiology: atrial fibrillation in cancer. J Am Coll Cardiol. (2014) 63(10):945–53. 10.1016/j.jacc.2013.11.026 24361314

[10] Onaitis M D'Amico T Zhao Y O'Brien S Harpole D. Risk factors for atrial fibrillation after lung cancer surgery: analysis of the society of thoracic surgeons general thoracic surgery database. Ann Thorac Surg. (2010) 90(2):368–74. 10.1016/j.athoracsur.2010.03.100 20667313

[11] Mosarla RC Vaduganathan M Qamar A Moslehi J Piazza G Giugliano RP. Anticoagulation strategies in patients with cancer: JACC review topic of the week. J Am Coll Cardiol. (2019) 73(11):1336–49. 10.1016/j.jacc.2019.01.017 30898209 PMC7957366

[12] Hu YF Liu CJ Chang PM Tsao HM Lin YJ Chang SL Incident thromboembolism and heart failure associated with new-onset atrial fibrillation in cancer patients. Int J Cardiol. (2013) 165(2):355–7. 10.1016/j.ijcard.2012.08.036 22989607

[13] Kamphuisen PW Beyer-Westendorf J. Bleeding complications during anticoagulant treatment in patients with cancer. Thromb Res. (2014) 133(Suppl 2):S49–55. 10.1016/S0049-3848(14)50009-6 24862146

[14] Joglar JA Chung MK Armbruster AL Benjamin EJ Chyou JY Cronin EM 2023 Acc/Aha/Accp/Hrs guideline for the diagnosis and management of atrial fibrillation: a report of the American College of Cardiology/American Heart Association joint committee on clinical practice guidelines. Circulation. (2024) 149(1):e1–56. 10.1161/CIR.0000000000001193 38033089 PMC11095842

[15] Asnani A Manning A Mansour M Ruskin J Hochberg EP Ptaszek LM. Management of atrial fibrillation in patients taking targeted cancer therapies. Cardiooncology. (2017) 3:2. 10.1186/s40959-017-0021-y 32153998 PMC7048041

[16] Li Q Liu F Tang Y Lee S Lang C Bai L The distribution of cardiovascular-related comorbidities in different adult-onset cancers and related risk factors: analysis of 10 year retrospective data. Front Cardiovasc Med. (2021) 8:695454. 10.3389/fcvm.2021.695454 34595215 PMC8476781

[17] O'Neal WT Lakoski SG Qureshi W Judd SE Howard G Howard VJ Relation between cancer and atrial fibrillation (from the reasons for geographic and racial differences in stroke study). Am J Cardiol. (2015) 115(8):1090–4. 10.1016/j.amjcard.2015.01.540 25711434 PMC4380860

[18] Jakobsen CB Lamberts M Carlson N Lock-Hansen M Torp-Pedersen C Gislason GH Incidence of atrial fibrillation in different Major cancer subtypes: a nationwide population-based 12 year follow up study. BMC Cancer. (2019) 19(1):1105. 10.1186/s12885-019-6314-9 31726997 PMC6854796

[19] Kattelus H Kesaniemi YA Huikuri H Ukkola O. Cancer increases the risk of atrial fibrillation during long-term follow-up (opera study). PLoS One. (2018) 13(10):e0205454. 10.1371/journal.pone.0205454 30289944 PMC6173458

[20] Kanmanthareddy A Vallakati A Reddy Yeruva M Dixit S DI Biase L Mansour M Pulmonary vein isolation for atrial fibrillation in the postpneumonectomy population: a feasibility, safety, and outcomes study. J Cardiovasc Electrophysiol. (2015) 26(4):385–9. 10.1111/jce.12619 25588757

[21] Fradley MG Beckie TM Brown SA Cheng RK Dent SF Nohria A Recognition, prevention, and management of arrhythmias and autonomic disorders in cardio-oncology: a scientific statement from the American Heart Association. Circulation. (2021) 144(3):e41–55. 10.1161/CIR.0000000000000986 34134525 PMC8992663

[22] Thotamgari SR Sheth AR Patel HP Sandhyavenu H Patel B Grewal US Safety of catheter ablation for atrial fibrillation in patients with cancer: a nationwide cohort study. Postgrad Med. (2023) 135(6):562–8. 10.1080/00325481.2023.2218188 37224412

[23] Giustozzi M Ali H Reboldi G Balla C Foresti S de Ambroggi G Safety of catheter ablation of atrial fibrillation in cancer survivors. J Interv Card Electrophysiol. (2021) 60(3):419–26. 10.1007/s10840-020-00745-7 32377917

[24] Agarwal S Munir MB Krishan S Yang EH Barac A Asad ZUA. Outcomes and readmissions in patients with cancer undergoing catheter ablation for atrial fibrillation. Europace. (2023) 25(9). 10.1093/europace/euad263 PMC10485182 37655932

[25] Wu L Narasimhan B Yang Z Bhatia K Li P Shah A Abstract 15839: complication and readmission outcome of atrial fibrillation catheter ablation among cancer patients. Circulation. (2022) 146(Suppl_1):A15839–A. 10.1161/circ.146.suppl_1.15839

[26] Ganatra S Abraham S Kumar A Parikh R Patel R Khadke S Efficacy and safety of catheter ablation for atrial fibrillation in patients with history of cancer. Cardiooncology. (2023) 9(1):19. 10.1186/s40959-023-00171-4 37020260 PMC10074889

[27] Eitel C Sciacca V Bartels N Saraei R Fink T Keelani A Safety and efficacy of cryoballoon based pulmonary vein isolation in patients with atrial fibrillation and a history of cancer. J Clin Med. (2021) 10(16). 10.3390/jcm10163669 PMC8397043 34441965

[28] Peng X He L Liu N Ruan Y Zhao X Guo X Outcome of cancer patients after atrial fibrillation ablation: insights from the China-af registry. Pacing Clin Electrophysiol. (2023) 46(11):1419–29. 10.1111/pace.14830 37736690

[29] Wang YS Li DB Chen C Wei YS Lyu HC Han JY [Feasibility of radiofrequency ablation for cancer patients with atrial fibrillation]. Zhonghua Xin Xue Guan Bing Za Zhi. (2021) 49(8):790–5. 10.3760/cma.j.cn112148-20200922-00758 34404188

[30] Lopez-Fernandez T Martin-Garcia A Roldan Rabadan I Mitroi C Mazon Ramos P Diez-Villanueva P Atrial fibrillation in active cancer patients: expert position paper and recommendations. Rev Esp Cardiol (Engl Ed). (2019) 72(9):749–59. 10.1016/j.recesp.2019.03.017 31405794

[31] Lyon AR Lopez-Fernandez T Couch LS Asteggiano R Aznar MC Bergler-Klein J 2022 Esc guidelines on cardio-oncology developed in collaboration with the European Hematology Association (Eha), the European Society for Therapeutic Radiology and Oncology (Estro) and the International Cardio-Oncology Society (Ic-Os). Eur Heart J. (2022) 43(41):4229–361. 10.1093/eurheartj/ehac244 36017568

[32] Boriani G Lee G Parrini I Lopez-Fernandez T Lyon AR Suter T Anticoagulation in patients with atrial fibrillation and active cancer: an international survey on patient management. Eur J Prev Cardiol. (2021) 28(6):611–21. 10.1093/eurjpc/zwaa054 33624005

[33] Isogai T Saad AM Abushouk AI Shekhar S Kuroda S Gad MM Procedural and short-term outcomes of percutaneous left atrial appendage closure in patients with cancer. Am J Cardiol. (2021) 141:154–7. 10.1016/j.amjcard.2020.12.003 33279485

