# Supplementary material for: Selection of rhythm intervention strategies in atrial fibrillation patients with cancer and efficacy and safety of catheter ablation
Source: Front Cardiovasc Med. 2024 Nov 22;11:1506143. doi: 10.3389/fcvm.2024.1506143 (PMC11621071; doi:10.3389/fcvm.2024.1506143)
Supplement: Supplementary file 1 [file Table1.docx]

**Table S1. Baseline characteristics between AF patients with cancer and without cancer after PSM**

| **Variables** | **Total**  **(n=820)** | **No Cancer**  **(n=410)** | **Cancer**  **(n=410)** | ***P* value** | **SMD** |
| --- | --- | --- | --- | --- | --- |
| Age, years | 71.43 (9.35) | 71.68 (9.53) | 71.18 (9.18) | 0.442 | -0.055 |
| Male, n (%) | 439 (53.54)) | 228 (55.61) | 211 (51.46) | 0.234 | -0.083 |
| Hospital stays, days | 6.30 (4.23) | 6.40 (4.40) | 6.19 (4.06 | 0.476 | -0.052 |
| Smoking, n (%) | 129 (15.73) | 64 (15.61) | 65 (15.85) | 0.924 | 0.007 |
| Drinking, n (%) | 95 (11.59) | 46 (11.22) | 49 (11.95) | 0.743 | 0.023 |
| SBP, mmHg | 135.35 (19.18) | 136.00 (19.51) | 134.70 (18.85) | 0.331 | -0.069 |
| DBP, mmHg | 80.32 (13.10) | 80.20 (12.99) | 80.43 (13.23) | 0.804 | 0.017 |
| Hemoglobin count, g/L | 135.08 (17.32) | 135.72 (16.63) | 134.43 (17.98) | 0.284 | -0.072 |
| Platelet count, 🞨109/L | 192.76 (52.81) | 191.63 (49.24) | 193.89 (56.20) | 0.541 | 0.040 |
| NLR | 1.86 (1.62, 2.60) | 1.86 (1.64, 2.58) | 1.86 (1.59, 2.67) | 0.810 | 0.023 |
| TC, mmol/L | 4.47 (0.75) | 4.48 (0.76) | 4.47 (0.75) | 0.809 | -0.017 |
| TG, mmol/L | 1.34 (0.50) | 1.33 (0.46) | 1.35 (0.53) | 0.519 | 0.042 |
| LDL-C, mmol/L | 2.49 (0.54) | 2.48 (0.54) | 2.49 (0.55) | 0.768 | 0.020 |
| HDL-C, mmol/L | 1.19 (0.24) | 1.20 (0.23) | 1.18 (0.24) | 0.184 | -0.091 |
| ALT, IU/L | 23.75 (13.59) | 23.46 (12.72) | 24.03 (14.42) | 0.549 | 0.039 |
| AST, IU/L | 24.10 (11.16) | 23.85 (11.34) | 24.35 (10.98) | 0.515 | 0.046 |
| eGFR, ml/min/1.73m^2^ | 83.65 (24.46) | 83.21 (23.56) | 84.08 (25.34) | 0.609 | 0.035 |
| SUA, μmol/L | 358.07 (90.26) | 360.04 (89.21) | 356.11 (91.38) | 0.533 | -0.043 |
| K^+^, mmol/L | 4.04 (0.42) | 4.03 (0.41) | 4.05 (0.44) | 0.491 | 0.049 |
| Na^+^, mmol/L | 141.61 (2.88) | 141.69 (2.67) | 141.54 (3.08) | 0.473 | -0.047 |
| Mg^2+^, mmol/L | 0.92 (0.10) | 0.92 (0.10) | 0.92 (0.09) | 0.859 | 0.017 |
| FBG, mmol/L | 5.93 (2.10) | 5.97 (2.38) | 5.90 (1.79) | 0.704 | -0.040 |
| Coagulation indicators |  |  |  |  |  |
| TT, s | 21.28 (19.09) | 21.33 (19.60) | 21.23 (18.63) | 0.943 | -0.005 |
| PT, s | 13.50 (5.59) | 13.51 (5.86) | 13.48 (5.33) | 0.931 | -0.007 |
| APTT, s | 29.04 (20.95) | 28.03 (7.15) | 30.00 (28.44) | 0.208 | 0.069 |
| Fib, g/L | 2.97 (0.81) | 2.95 (0.75) | 2.98 (0.87) | 0.559 | 0.038 |
| TSH, mIU/L | 2.88 (5.95) | 2.64 (3.55) | 3.12 (7.64) | 0.293 | 0.063 |
| BNP, ng/L | 199.18 (201.03) | 189.60 (184.40) | 208.77 (216.19) | 0.172 | 0.089 |
| Echocardiographic parameters |  |  |  |  |  |
| LAD, mm | 40.84 (6.25) | 41.14 (6.80) | 40.55 (5.64) | 0.266 | -0.104 |
| LVEDD, mm | 47.99 (5.72) | 48.34 (6.11) | 47.64 (5.29) | 0.148 | -0.131 |
| LVEF, % | 55.22 (7.28) | 54.83 (8.01) | 55.61 (6.46) | 0.203 | 0.121 |
| Previous history, n (%) |  |  |  |  |  |
| HTN | 529 (64.51) | 266 (64.88) | 263 (64.15) | 0.827 | -0.015 |
| CHD | 325 (39.63) | 190 (46.34) | 135 (32.93) | <0.001 | -0.285 |
| HF | 234 (28.54) | 117 (28.54) | 117 (28.54) | 1.000 | 0.000 |
| Stroke | 102 (12.44) | 54 (13.17) | 48 (11.71) | 0.526 | -0.046 |
| PAD | 7 (0.85) | 4 (0.98) | 3 (0.73) | 1.000 | -0.029 |
| Dyslipidemia | 513 (62.56) | 265 (64.63) | 248 (60.49) | 0.220 | -0.085 |
| T2DM | 214 (26.1) | 107 (26.10) | 107 (26.10) | 1.000 | 0.000 |
| CKD | 124 (15.12) | 66 (16.10) | 58 (14.15) | 0.436 | -0.056 |
| Hyperthyroidism | 17 (2.07) | 8 (1.95) | 9 (2.20) | 0.806 | 0.017 |
| Hypothyroidism | 11 (1.34) | 4 (0.98) | 7 (1.71) | 0.362 | 0.056 |
| VTE | 6 (0.73) | 2 (0.49) | 4 (0.98) | 0.682 | 0.050 |
| COPD | 6 (0.73) | 3 (0.73) | 3 (0.73) | 1.000 | 0.000 |
| Types of AF, n (%) |  |  |  | 0.777 |  |
| PaAF | 474 (57.8) | 235 (57.32) | 239 (58.29) |  | 0.020 |
| PeAF | 346 (42.2) | 175 (42.68) | 171 (41.71) |  | -0.020 |
| Antiarrhythmic drugs, n (%) |  |  |  | 0.902 |  |
| Amiodarone | 203 (24.76) | 104 (25.37) | 99 (24.15) |  | -0.028 |
| Propafenone | 137 (16.71) | 69 (16.83) | 68 (16.59) |  | -0.007 |
| β-blocker | 480 (58.54) | 237 (57.80) | 243 (59.27) |  | 0.030 |
| Anticoagulants, n (%) |  |  |  | 0.944 |  |
| Warfarin | 405 (49.39) | 202 (49.27) | 203 (49.51) |  | 0.005 |
| NOACs | 415 (50.61) | 208 (50.73) | 207 (50.49) |  | -0.005 |
| AF radiofrequency ablation, n (%) | 195 (23.78) | 102 (24.88) | 93 (22.68) | 0.460 | -0.052 |

Abbreviations: AF, atrial fibrillation; ALT, alanine transaminase; APTT, activated partial thromboplastin time; AST, aspartate aminotransferase; BNP, B-type natriuretic peptide; CHD, coronary heart disease; CKD, chronic kidney disease; COPD, chronic obstructive pulmonary disease; DBP, diastolic blood pressure; eGFR, estimated glomerular filtration rate; FBG, fasting blood glucose; Fib, plasma fibrinogen; HDL-C, high-density lipoprotein cholesterol; HF, heart failure; HTN, hypertension; LAD, left atrial diameter; LDL-C, low-density lipoprotein cholesterol; LVEDD, left ventricular end diastolic diameter; LVEF, left ventricular ejection fraction; NLR, neutrophil to lymphocyte ratio; NOACs, non-vitamin K antagonist oral anticoagulants; PaAF, paroxysmal atrial fibrillation; PAD, peripheral arterial disease; PeAF, persistent atrial fibrillation; PSM, Propensity score matching; PT, prothrombin time; SBP, systolic blood pressure; SMD, standardized mean difference; SUA, serum uric acid; T2DM, type2 diabetes mellitus; TC, total cholesterol; TG, triglyceride; TT, thrombin time; TSH, thyroid stimulating hormone; VTE, venous thromboembolism

**TableS2. Factors associated with ablation in AF patients after PSM**

| **Variables** | **Univariate analysis** | | **Multivariate analysis** | |
| --- | --- | --- | --- | --- |
|  | **OR (95%CI)** | ***P* value** | **OR (95%CI)** | ***P* value** |
| Age | 0.92 (0.91 - 0.94) | <0.001 | 0.93 (0.90 - 0.96) | <0.001 |
| TSH | 0.90 (0.82 - 0.99) | 0.039 | 0.89 (0.78 - 1.01) | 0.067 |
| LAD | 0.93 (0.90 - 0.96) | <0.001 | 0.94 (0.90 - 0.99) | 0.018 |
| Male | 0.82 (0.59 - 1.13) | 0.224 |  |  |
| HTN | 0.60 (0.43 - 0.84) | 0.002 | 1.09 (0.68 - 1.76) | 0.724 |
| CHD | 0.50 (0.35 - 0.71) | <0.001 | 0.62 (0.38 - 1.02) | 0.059 |
| HF | 0.31 (0.20 - 0.48) | <0.001 | 0.73 (0.38 - 1.39) | 0.335 |
| Stroke | 0.39 (0.21 - 0.73) | 0.003 | 0.43 (0.18 - 1.04) | 0.060 |
| PAD | 1.28 (0.25 - 6.68) | 0.766 |  |  |
| T2DM | 0.52 (0.35 - 0.79) | 0.002 | 0.56 (0.31 - 0.99) | 0.046 |
| CKD | 0.24 (0.12 - 0.47) | <0.001 | 0.57 (0.25 - 1.27) | 0.169 |
| VTE | 0.64 (0.07 - 5.50) | 0.684 |  |  |
| PeAF | 0.61 (0.44 - 0.86) | 0.004 | 0.98 (0.58 - 1.66) | 0.940 |
| Cancer | 0.89 (0.64 - 1.22) | 0.460 |  |  |

Abbreviations: see Table S1; CI, confidence interval; OR, odds ratio

**Table S3. Comparison of baseline characteristics between cancer patients who receive ablation and those who do not receive ablation**

| **Variables** | **Cancer patients**  **(n=410)** | **Without ablation**  **(n=317)** | **With ablation**  **(n=93)** | ***P* value** |
| --- | --- | --- | --- | --- |
| Age, years | 71.18 (9.18) | 72.69 (8.99) | 66.00 (7.84) | <0.001 |
| Male, n (%) | 211 (51.46) | 172 (54.26) | 39 (41.94) | 0.005 |
| Hospital stays, days | 6.19 (4.06) | 5.57 (3.50) | 8.31 (5.02) | <0.001 |
| Smoking, n (%) | 65 (15.85) | 50 (15.77) | 15 (16.13) | 0.934 |
| Drinking, n (%) | 49 (11.95) | 36 (11.36) | 13 (13.98) | 0.493 |
| SBP, mmHg | 134.70 (18.85) | 134.71 (18.96) | 134.63 (18.58) | 0.971 |
| DBP, mmHg | 80.43 (13.23) | 80.10 (14.07) | 81.57 (9.83) | 0.255 |
| Hemoglobin count, g/L | 134.43 (17.98) | 133.85 (18.86) | 136.40 (14.48) | 0.167 |
| Platelet count, 🞨109/L | 193.89 (56.20) | 191.96 (59.00) | 200.45 (45.00) | 0.140 |
| NLR | 1.86 (1.59 - 2.67) | 1.98 (1.67 - 2.90) | 1.84 (1.41 - 1.95) | <0.001 |
| TC, mmol/L | 4.47 (0.75) | 4.44 (0.73) | 4.55 (0.80) | 0.215 |
| TG, mmol/L | 1.35 (0.53) | 1.30 (0.44) | 1.52 (0.75) | 0.007 |
| LDL-C, mmol/L | 1.18 (0.24) | 1.18 (0.24) | 1.19 (0.24) | 0.714 |
| HDL-C, mmol/L | 2.49 (0.55) | 2.48 (0.53) | 2.52 (0.61) | 0.553 |
| ALT, IU/L | 24.03 (14.42) | 24.27 (15.41) | 23.23 (10.42) | 0.543 |
| AST, IU/L | 24.35 (10.98) | 25.28 (11.92) | 21.18 (5.90) | <0.001 |
| eGFR, ml/min/1.73m^2^ | 84.08 (25.34) | 82.29 (26.61) | 90.18 (19.34) | 0.002 |
| SUA, μmol/L | 356.11 (91.38) | 356.40 (91.07) | 355.10 (92.91) | 0.904 |
| K^+^, mmol/L | 4.05 (0.44) | 4.06 (0.46) | 4.03 (0.36) | 0.575 |
| Na^+^, mmol/L | 141.54 (3.08) | 141.37 (3.20) | 142.13 (2.54) | 0.036 |
| Mg^2+^, mmol/L | 0.92 (0.09) | 0.92 (0.09) | 0.93 (0.09) | 0.292 |
| FBG, mmol/L | 5.90 (1.79) | 5.95 (1.88) | 5.75 (1.48) | 0.449 |
| Coagulation indicators |  |  |  |  |
| TT, s | 21.23 (18.63) | 20.97 (18.83) | 22.18 (18.00) | 0.605 |
| PT, s | 13.48 (5.33) | 13.32 (4.74) | 14.02 (7.05) | 0.297 |
| APTT, s | 30.00 (28.44) | 30.49 (31.98) | 28.25 (7.01) | 0.532 |
| Fib, g/L | 2.98 (0.87) | 3.04 (0.94) | 2.78 (0.56) | <0.001 |
| TSH, mIU/L | 3.12 (7.64) | 3.36 (8.61) | 2.28 (1.66) | 0.277 |
| BNP, ng/L | 208.77 (216.19) | 224.92 (239.63) | 153.75 (81.16) | <0.001 |
| Echocardiographic parameters |  |  |  |  |
| LAD, mm | 40.55 (5.64) | 40.80 (5.87) | 39.82 (4.83) | 0.205 |
| LVEDD, mm | 47.64 (5.29) | 47.57 (5.57) | 47.86 (4.39) | 0.689 |
| LVEF, % | 55.61 (6.46) | 55.24 (6.83) | 56.69 (5.05) | 0.102 |
| Previous history, n (%) |  |  |  |  |
| HTN | 263 (64.15) | 209 (65.93) | 54 (58.06) | 0.164 |
| CHD | 135 (32.93) | 115 (36.28) | 20 (21.51) | 0.008 |
| HF | 117 (28.54) | 103 (32.49) | 14 (15.05) | 0.001 |
| Stroke | 48 (11.71) | 41 (12.93) | 7 (7.53) | 0.154 |
| PAD | 3 (0.73) | 2 (0.63) | 1 (1.08) | 0.539 |
| Dyslipidemia | 248 (60.49) | 195 (61.51) | 53 (56.99) | 0.433 |
| T2DM | 107 (26.1) | 90 (28.39) | 17 (18.28) | 0.051 |
| CKD | 58 (14.15) | 53 (16.72) | 5 (5.38) | 0.006 |
| Hyperthyroidism | 58 (14.15) | 53 (16.72) | 5 (5.38) | 0.006 |
| Hypothyroidism | 7 (1.71) | 6 (1.89) | 1 (1.08) | 0.936 |
| VTE | 4 (0.98) | 3 (0.95) | 1 (1.08) | 1.000 |
| COPD | 3 (0.73) | 3 (0.95) | 0 (0.00) | 1.000 |
| Types of AF, n (%) |  |  |  | 0.019 |
| PaAF | 239 (58.29) | 175 (55.21) | 64 (68.82) |  |
| PeAF | 171 (41.71) | 142 (44.79) | 29 (31.18) |  |
| Antiarrhythmic drugs, n (%) |  |  |  | <0.001 |
| Amiodarone | 99 (24.15) | 39 (12.30) | 60 (64.52) |  |
| Propafenone | 68 (16.59) | 50 (15.77) | 18 (19.35) |  |
| β-blocker | 243 (59.27) | 228 (71.92) | 15 (16.13) |  |
| Anticoagulants, n (%) |  |  |  | <0.001 |
| Warfarin | 203 (49.51) | 178 (56.15) | 25 (26.88) |  |
| NOACs | 207 (50.49) | 139 (43.85) | 68 (73.12) |  |
| Active cancer, n (%) | 42 (10.24) | 33 (10.41) | 9 (9.68) | 0.838 |
| Anticancer therapy, n (%) |  |  |  |  |
| surgery | 375 (91.46) | 286 (90.22) | 89 (95.70) | 0.096 |
| chemotherapy | 44 (10.73) | 34 (10.73) | 10 (10.75) | 0.994 |
| radiotherapy | 20 (4.88) | 17 (5.36) | 3 (3.23) | 0.570 |

Abbreviations: see Table S1

**Table S4. Baseline characteristics between patients with and without cancer undergoing ablation after PSM**

| **Variables** | **Total**  **（n=184）** | **No cancer**  **（n=92）** | **Cancer**  **（n=92）** | ***P* value** | **SMD** |
| --- | --- | --- | --- | --- | --- |
| Age, years | 66.05 (7.93) | 66.11 (8.03) | 66.00 (7.88) | 0.926 | -0.014 |
| Male, n (%) | 76 (41.3) | 38 (41.30) | 38 (41.30) | 1.000 | 0.000 |
| Hospital stays, days | 7.96 (4.73) | 7.62 (4.39) | 8.30 (5.05) | 0.328 | 0.136 |
| Smoking, n (%) | 32 (17.39) | 17 (18.48) | 15 (16.30) | 0.697 | -0.059 |
| Drinking, n (%) | 23 (12.5) | 10 (10.87) | 13 (14.13) | 0.504 | 0.094 |
| SBP, mmHg | 134.67 (18.80) | 134.97 (19.19) | 134.37 (18.50) | 0.830 | -0.032 |
| DBP, mmHg | 80.94 (10.22) | 80.62 (11.00) | 81.26 (9.42) | 0.670 | 0.069 |
| Hemoglobin count, g/L | 137.15 (13.01) | 137.24 (13.00) | 137.05 (13.10) | 0.924 | -0.014 |
| Platelet count, 🞨109/L | 202.83 (41.89) | 204.59 (38.86) | 201.07 (44.85) | 0.570 | -0.079 |
| NLR | 1.99 (1.10) | 2.11 (1.39) | 1.86 (0.69) | 0.113 | -0.375 |
| TC, mmol/L | 4.57 (0.79) | 4.55 (0.84) | 4.58 (0.74) | 0.769 | 0.046 |
| TG, mmol/L | 1.47 (0.63) | 1.40 (0.47) | 1.53 (0.75) | 0.156 | 0.175 |
| LDL-C, mmol/L | 2.55 (0.61) | 2.56 (0.65) | 2.54 (0.57) | 0.851 | -0.030 |
| HDL-C, mmol/L | 1.21 (0.23) | 1.22 (0.22) | 1.19 (0.24) | 0.428 | -0.113 |
| ALT, IU/L | 22.91 (10.92) | 22.41 (11.51) | 23.41 (10.34) | 0.537 | 0.097 |
| AST, IU/L | 21.41 (6.21) | 21.52 (6.59) | 21.29 (5.83) | 0.797 | -0.040 |
| eGFR, ml/min/1.73m^2^ | 89.01 (18.90) | 87.42 (18.75) | 90.60 (19.03) | 0.256 | 0.167 |
| SUA, μmol/L | 343.58 (80.05) | 336.35 (75.99) | 350.82 (83.69) | 0.221 | 0.173 |
| K^+^, mmol/L | 4.02 (0.38) | 4.00 (0.40) | 4.03 (0.36) | 0.569 | 0.097 |
| Na^+^, mmol/L | 142.22 (2.43) | 142.27 (2.34) | 142.17 (2.52) | 0.799 | -0.036 |
| Mg^2+^, mmol/L | 0.94 (0.09) | 0.95 (0.09) | 0.94 (0.09) | 0.278 | -0.204 |
| FBG, mmol/L | 5.59 (1.46) | 5.39 (1.42) | 5.77 (1.48) | 0.157 | 0.261 |
| Coagulation indicators |  |  |  |  |  |
| TT, s | 22.82 (20.64) | 23.51 (23.39) | 22.22 (18.11) | 0.706 | -0.071 |
| PT, s | 13.46 (5.95) | 12.82 (4.26) | 14.03 (7.09) | 0.213 | 0.171 |
| APTT, s | 28.24 (7.21) | 28.35 (7.51) | 28.14 (6.98) | 0.861 | -0.030 |
| Fib, g/L | 2.80 (0.54) | 2.81 (0.53) | 2.79 (0.56) | 0.769 | -0.042 |
| TSH, mIU/L | 2.34 (1.61) | 2.41 (1.56) | 2.28 (1.67) | 0.616 | -0.081 |
| BNP, ng/L | 160.97 (120.68) | 170.15 (151.06) | 151.78 (79.35) | 0.303 | -0.231 |
| Echocardiographic parameters |  |  |  |  |  |
| LAD, mm | 39.29 (4.78) | 38.74 (4.68) | 39.84 (4.86) | 0.175 | 0.227 |
| LVEDD, mm | 47.52 (4.34) | 47.16 (4.26) | 47.87 (4.42) | 0.335 | 0.161 |
| LVEF, % | 56.58 (4.93) | 56.49 (4.80) | 56.67 (5.09) | 0.825 | 0.037 |
| Previous history, n (%) |  |  |  |  |  |
| HTN | 108 (58.7) | 54 (58.70) | 54 (58.70) | 1.000 | 0.000 |
| CHD | 56 (30.43) | 37 (40.22) | 19 (20.65) | 0.004 | -0.483 |
| HF | 31 (16.85) | 17 (18.48) | 14 (15.22) | 0.555 | -0.091 |
| Stroke | 16 (8.7) | 9 (9.78) | 7 (7.61) | 0.601 | -0.082 |
| PAD | 1 (0.54) | 0 (0.00) | 1 (1.09) | 1.000 | 0.105 |
| Dyslipidemia | 118 (64.13) | 66 (71.74) | 52 (56.52) | 0.031 | -0.307 |
| T2DM | 33 (17.93) | 16 (17.39) | 17 (18.48) | 0.848 | 0.028 |
| CKD | 11 (5.98) | 7 (7.61) | 4 (4.35) | 0.351 | -0.160 |
| Hyperthyroidism | 2 (1.09) | 0 (0.00) | 2 (2.17) | 0.477 | 0.149 |
| Hypothyroidism | 2 (1.09) | 1 (1.09) | 1 (1.09) | 1.000 | 0.000 |
| VTE | 1 (0.54) | 0 (0.00) | 1 (1.09) | 1.000 | 0.105 |
| COPD | 1 (0.54) | 1 (1.09) | 0 (0.00) | 1.000 | -0.148 |
| Types of AF, n (%) |  |  |  | 0.243 |  |
| PaAF | 135 (73.37) | 71 (77.17) | 64 (69.57) |  | -0.165 |
| PeAF | 49 (26.63) | 21 (22.83) | 28 (30.43) |  | 0.165 |
| Antiarrhythmic drugs, n (%) |  |  |  | 0.188 |  |
| Amiodarone | 113 (61.41) | 53 (57.61) | 60 (65.22) |  | 0.160 |
| Propafenone | 38 (20.65) | 24 (26.09) | 14 (15.22) |  | -0.303 |
| β -blocker | 33 (17.93) | 15 (16.30) | 18 (19.57) |  | 0.082 |
| Anticoagulants, n (%) |  |  | 0.737 |  |  |
| Warfarin | 48 (26.09) | 23 (25.00) | 25 (27.17) |  | 0.049 |
| NOACs | 136 (73.91) | 69 (75.00) | 67 (72.83) |  | -0.049 |

Abbreviations: see Table S1
